# Restrictive right ventricular performance assessed by cardiac magnetic resonance after balloon valvoplasty of severe pulmonary stenosis in adolescents

**DOI:** 10.1186/1532-429X-14-S1-P102

**Published:** 2012-02-01

**Authors:** Ikram E Massoud, Elham Mohamed, Atef Yahia, Nader Botros, Ayman M Khalifa, Mohamed A Donya

**Affiliations:** 1 Cardiology, national heart institute, Cairo, Egypt; 2 Imaging Departement, Kasr El-Eini, Cairo, Egypt; 3 Biomedical Engineering, Helwan University, Cairo, Egypt

## Background

The fundamental abnormality of restrictive right ventricle (RV) physiology is reduced RV compliance as a result of chronic increase in pressure overload.

In this study, we test the hypothesis that the persistence of Restrictive RV physiology after balloon pulmonary valvoplasty (BPV) is related to RV fibrosis assessed by Gadolinium-DTPA delayed-enhancement magnetic resonance imaging (de-MRI).

## Methods

A total of 10 selected patients (17± 5) years with severe Pulmonary Valve Stenosis (PVS) referred for BPV were studied. All patients were cyanotic. 5 out of 10 patients presented with frank right sided failure. Restrictive RV physiology was assessed by Doppler echocardiography defined by the presence of antegrade diastolic pulmonary flow.

All patients had undergone successful BPV with a recorded hemodynamics. Their RV function (lateral tricuspid annulus motion) long-axis movement was measured by M-mode and Pulsed Wave (PW) Tissue Doppler imaging (TDI) during the follow up period range from (1-5) years. Also their RV function was compared with 10 normal controls. The RV fibrosis for these patients was assessed using de-MRI.

## Results

### Hemodynamic

Before BPV, RV Angiogram demonstrate severe RV hypertrophy in all patients. RV hypertrophy and dilatation with impaired systolic function in 5 out of 10 patients. RV end diastolic pressure mean 20±8 was significantly higher than pulmonary end diastolic pressure mean 12±8mmHg P≤0.001.

After BPV, the RV systolic pressure decreased from mean (179±40mmHg) to (60±20mmHg) with significant P<0.001. RV end diastolic pressure decreased from mean (20±8 mmHg) to (14±6mmHg) with significant P<0.001 after BPV.

Compared with control group patients with restrictive physiology, patients had a lower tricuspid annular systolic and diastolic velocities and lower RV long-axis function.

### Tissue characterization

During follow up, delayed enhancement MRI revealed the presence of varying degrees of myocardial fibroses and scarring which ranged from mild patchy areas of enhancement seen at the insertion points, to full enhancement of anterior right ventricular free wall, explaining the presence of restrictive RV physiology in those patients.

## Conclusions

persistence of restrictive RV physiology after relief of pressure overload in severe pulmonary valve stenosis in adolescents reflects RV diastolic dysfunction associated with RV fibrosis.

## Funding

Egyptian Medical Insurance.

